# Design of NiO Flakes@CoMoO_4_ Nanosheets Core-Shell Architecture on Ni Foam for High-Performance Supercapacitors

**DOI:** 10.1186/s11671-019-3054-3

**Published:** 2019-07-02

**Authors:** Enmin Zhou, Liangliang Tian, Zhengfu Cheng, Chunping Fu

**Affiliations:** 1 Research Institute for New Materials Technology, Chongqing University of Arts and Sciences, Chongqing, People’s Republic of China; 2 School of Science, Chongqing University of Posts and Telecommunications, Chongqing, People’s Republic of China

**Keywords:** Transition metal oxide, Core-shell structure, Hydrothermal method, Energy storage, Pseudocapacitors

## Abstract

As typical electrode materials for supercapacitors, low specific capacitance and insufficient cycling stability of transition metal oxides (TMOs) are still the problems that need to be solved. Design of core-shell structure is considered as an effective method for preparation of high-performance electrode materials. In this work, NiO flakes@CoMoO_4_ nanosheets/Ni foam (NiO flakes@CoMoO_4_ NSs/NF) core-shell architecture was constructed by a two-step hydrothermal method. Interestingly, the CoMoO_4_ NSs are vertically grown on the surface of NiO flakes, forming a two-dimensional (2D) branched core-shell structure. The porous core-shell architecture has relatively high surface area, effective ions channels, and abundant redox sites, resulting in excellent electrochemical performance. As a positive electrode for supercapacitors, NiO flakes@CoMoO_4_ NSs/NF core-shell architecture exhibits excellent capacitive performance in terms of high specific capacitance (1097 F/g at 1 A/g) and outstanding cycling stability (97.5% after 2000 circles). The assembled asymmetric supercapacitor (ASC) of NiO flakes@CoMoO_4_ NSs/NF//active carbon (AC)/NF possesses a maximum energy density of 25.8 Wh/kg at power density of 894.7 W/kg. The results demonstrate that NiO flakes@CoMoO_4_ NSs/NF electrode displays potential applications in supercapacitors and the design of 2D branched core-shell architecture paves an ideal way to obtain high-performance TMOs electrodes.

**Electronic supplementary material:**

The online version of this article (10.1186/s11671-019-3054-3) contains supplementary material, which is available to authorized users.

## Introduction

Currently, the requirements for renewable energy resources and energy storage devices are being increased rapidly with the fast development of technology and the social progress [1, 2]. The properties of fast charge-discharge rate, better safety feature, high power density, and long-life span make supercapacitors become one of the most promising candidates for traditional energy storage devices. According to storage mechanism, supercapacitors are generally classified into two types, including electric double-layer capacitors (EDLCs) and pseudocapacitors [3]. EDLCs store charge by the way of electrostatic adsorption on the interface of electrode/electrolyte. Pseudocapacitors store energy by the redox reactions (or underpotential deposition and intercalation), which happens onto/near the surface of electrode materials [4, 5]. Thereinto, the pseudocapacitors have become the focus of research because of the higher energy density compared to EDLCs.

Transition metal oxides (TMOs) have been taken into consideration as electrode materials for pseudocapacitors owing to high theory specific capacitance, nature abundant, low cost, and environment friendly [6, 7]. Whereas the obtained experiment value of specific capacitance is much smaller than the value of theory specific capacitance due to the incomplete utilization of electrode materials [8]. Furthermore, TMOs electrode always shows insufficient stability during the charge-discharge process because of the continuous change of volume [9]. Usually, there are two effective methods to solve the above-mentioned problems. On the one hand, directly growing electrode materials onto collector is beneficial to avoid the formation of “dead surface,” leading to the improvements of utilization [10]. Furthermore, the collector can apparently enhance the electrical conductivity of the electrode. On the other hand, inspired by kinetics, the design and tailoring of microstructures of electrode materials are considered as meaningful ideal to improve the capacitive performance. Researchers have constructed lots of electrode materials with different microstructures [11]. Thereinto, superior capacitive performance can be achieved through the design of core-shell architecture. This can be ascribed to the synergistic effect between band structure and electronic states density of core and shell materials [12–14]. Moreover, the core materials accelerate the electron transfer rate and the shell materials provide adequate electrochemical redox active sites. However, the traditional core-shell structure with “egg” model exists significant defect that the wrapped core materials cannot be effectively utilized due to the shielding of the shell materials. Therefore, the improvement of utilization for core materials is key for the capacitive performance of core-shell TMOs electrode.

In this work, a novel two-dimensional (2D) branched core-shell structure of NiO flakes@CoMoO_4_ nanosheets (NSs) was constructed by a two-step hydrothermal method to solve the mentioned drawbacks. Regarding this novel structure, uniform CoMoO_4_ NSs are vertically deposited onto the NiO flakes, forming a flakes-nanosheets core-shell structure. This 2D branched core-shell structure possesses the following advantages: first, the 2D branched core-shell structure affords enough contacting areas between electrolyte and electrode materials, providing sufficient electroactive sites; secondly, the 2D feature of NiO flakes and CoMoO_4_ NSs improve the electron collection efficiency and fasten the electron transfer rate, guaranteeing the advantages in electron transfer kinetics; and thirdly, the diffusion channels formed through the interaction of CoMoO_4_ NSs accelerate the diffusion of electrolyte, which are salutary for the utilization of core materials. In addition, the highly porous architecture provides interspaces for the release of stress formed during the charge-discharge process, further guaranteeing the cycling stability. In view of the above advantages, the NiO flakes@CoMoO_4_ NSs/NF electrode exhibits excellent electrochemical performance in terms of high specific capacitance of 1097 F/g and long cycling stability (retains 97.5% of original specific capacitance after 2000 cycles). The assembled asymmetric supercapacitors (ASCs) of NiO flakes@CoMoO_4_ NSs/NF//AC/NF have a high energy density of 25.8 Wh/kg at a power density of 894.7 W/kg. The results demonstrate that NiO flakes@CoMoO_4_ NSs has potential applications in energy storage device and the building of 2D branched structure paves an ideal way to achieve high-performance TMOs electrode materials.

## Methods Section

### Synthesis of NiO Flakes/NF

**Fig. 1:**
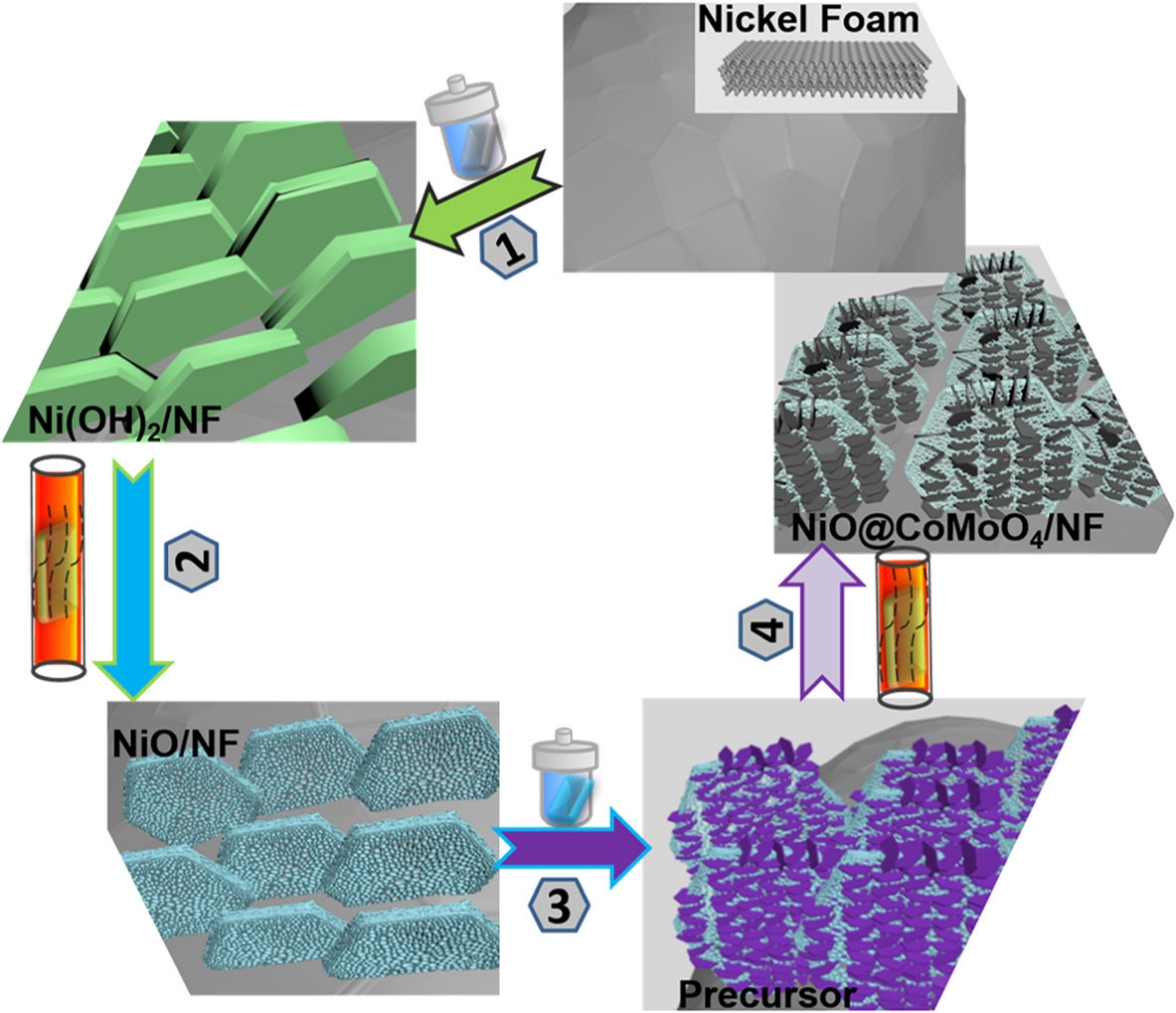
The synthesis illustration of NiO flakes@CoMoO_4_ NSs electrode

All chemicals used in this work were purchased from Aladdin reagent and used directly. The preparation flow diagram of electrode materials was shown in Fig. 1. A piece of NF (1.5 × 3.5 cm^2^) was immersed into 3 M HCl for 2 h to remove the oxide layer and dried at 60 °C for 12 h. Then, the pretreated NF was immersed into 32 mL distilled water and transferred into a 40 mL stainless steel autoclave. Subsequently, the autoclave was sealed and maintained at 140 °C for 24 h and naturally cooled to room temperature (step 1). The products were washed by deionized water for several times and dried in a vacuum chamber at 60 °C for 24 h. Furthermore, the prepared products were annealed in quartz tube furnace at 400 °C for 2 h with a heating rate of 0.5 °C/min (step 2).

### Synthesis of NiO Flakes@CoMoO_4_ NSs/NF

Cobalt chloride hexahydrate (65.1 mg) (CoCl_2_·6H_2_O) and sodium molybdate dihydrate (50.8 mg) (Na_2_MoO_4_·2H_2_O) were dispersed into 23 mL deionized water under stirring. Then the prepared NiO flakes/NF was immersed into the mentioned solution for 30 min and transferred into a 40 mL stainless autoclave. After that, the autoclave was maintained at 160 °C for 6 h and cooled to room temperature (step 3). The products were treated by ultrasonic for 2 min in deionized water to remove the loosely adsorbed chemicals and dried in vacuum at 60 °C for 12 h. Finally, NiO flakes@CoMoO_4_ NSs/NF was obtained by calcination at 400 °C for 2 h with a heating rate of 0.5 °C/min in quartz tube furnace (step 4). CoMoO_4_ flakes/NF was prepared through the same process using NF instead of NiO flakes/NF.

### Materials Characterization

The crystal structure of the products was characterized via X-ray diffractometer (XRD, Rigaku D/Max-02400) using Cu K_α_ radiation (1.54056 Å) with the work potential of 20 kV and tube current 30 mA. The field emission scanning electron microscopy (FESEM) images were obtained with the Ziess Gemini and Hitachi SU8100 at operating voltage of 5 kV and 3 kV, respectively. The high-resolution transmission electron microscope (HRTEM) observations were conducted on a JEM-2100F equipment. X-ray photoelectron spectroscopy (XPS) data were recorded on a Thermo ESCALAB 250Xi device at 200 kV. The specific surface area and pore distribution of the products were collected by BELSORP-max using high pure N_2_ as the absorption gas at temperature of 77 K.

### Electrochemical Measurements

All the electrochemical tests were performed via a μIII Autolab workstation with three electrode system in 6 M KOH, including saturated Ag/AgCl as reference electrode, platinum foil (1 cm × 1 cm) as counter electrode and NiO flakes@CoMoO_4_ NSs/NF (CoMoO_4_ flakes/NF or NiO flakes/NF) as working electrodes (1 cm × 1 cm). The capacitive performance was evaluated by galvanostatic charge-discharge (GCD) and cyclic voltammetry (CV) methods. The electrochemical impedance spectroscopy (EIS) data were collected in the frequency range from 100 kHz to 0.01 Hz under ambient condition. The mass loading of NiO flakes on NF was reasoned by evaluating the lost H_2_O in the decomposition process of Ni(OH)_2_, Eq. (1).

1 $$ m\left(\mathrm{NiO}\right)\kern0.5em =\kern0.5em \frac{M\left(\mathrm{NiO}\right)}{M\left({\mathrm{H}}_2\mathrm{O}\right)}\kern0.5em \times \kern0.5em m\left({\mathrm{H}}_2\mathrm{O}\right) $$

Where *m* and *M* represent the mass of each single materials and relative molecular mass, respectively. The mass loading of CoMoO_4_ NSs on NiO flakes/NF was obtained by calculating the mass difference before the second-step hydrothermal treatment and after the second post-calcination. The mass loading of CoMoO_4_ flakes/NF was calculated by evaluating the mass difference before and after the preparation. The mass loading of NiO flakes and CoMoO_4_ NSs directly on NF is 0.79 mg/cm^2^ and 1.14 mg/cm^2^, respectively. The mass loading of NiO flakes@CoMoO_4_ NSs/NF is 1.93 mg/cm^2^.

The electrochemical performance of ASCs was measured using two electrode system in 6 M KOH. Thereinto, NiO flakes@CoMoO_4_ NSs/NF, NiO flakes/NF, and CoMoO_4_ flakes/NF were applied as positive electrodes. The negative electrodes were synthesized by casting the mixture containing commercial active carbon, acetylene black, and polytetrafluoroethylene (PTFE) (mass ratio is 8:1:1) onto the surface of NF. The mass of active carbon (AC) is calculated according to Eq. (2) [15].

2 $$ \frac{m_{+}}{m_{-}}=\frac{C_{-}\times \varDelta {V}_{-}}{C_{+}\times \varDelta {V}_{+}} $$

Where *C* (F/g) is the specific capacitance, *∆V* (V) is the voltage window, and *m* (g) is the mass of electrode materials.

## Results and Discussion

### Characterizations

**Fig. 2:**
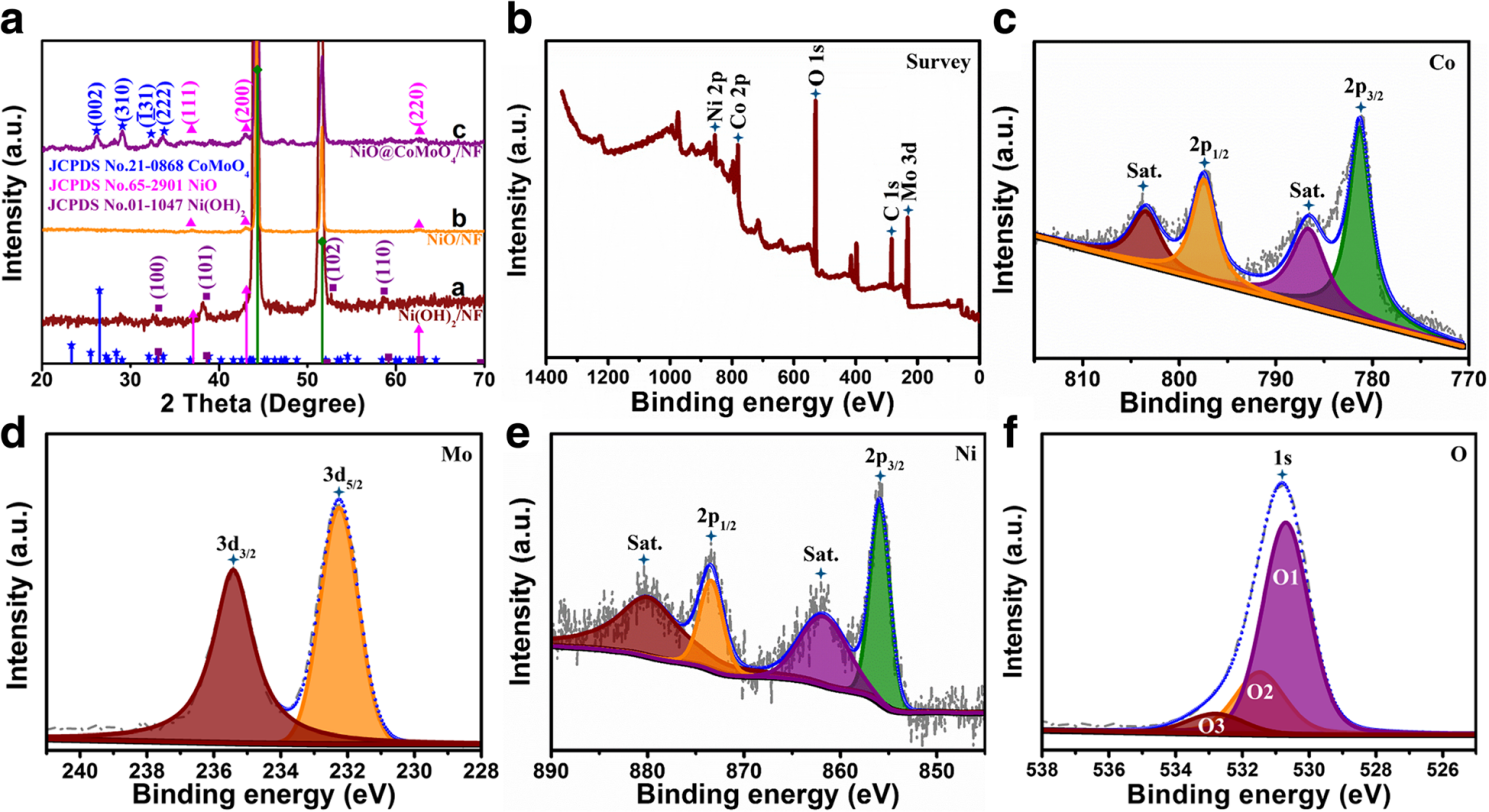
**a** The XRD patterns of Ni(OH)_2_ flakes/NF (curve a), NiO flakes/NF (curve b), and NiO flakes@CoMoO_4_ NSs/NF (curve c). The XPS spectra of NiO flakes@CoMoO_4_ NSs/NF. **b** Survey. **c** Co 2p. **d** Mo 3d. **e** Ni 2p. **f** O 1 s

The phase structure of the prepared samples was confirmed by XRD. As shown in Fig. 2a, the two strong diffraction peaks located at 44.3° and 51.7° can be assigned to characteristic of Ni (JCPDS No. 65-0380). After the first-step hydrothermal treatment, series of new diffraction peaks were investigated in curve a. The significant peaks can be indexed to the standard card of JCPDS No. 01-1047, indicating the formation of hexagonal *β*-Ni(OH)_2_ on NF. After the heat treatment at 400 °C, new diffraction peaks are observed in curve b; the formed new peaks are attributed to NiO (JCPDS No. 65-2901), indicating the decomposition of *β*-Ni(OH)_2_. Curve c displays the XRD pattern of final products. Apart from the diffraction peaks of NiO, the peaks at 26.5°, 29.1°, 32.1°, 33.7° agree well with (002), (310), ($\overline{1}31$), and ($\overline{2}22$) crystal planes of CoMoO_4_, respectively [16–18], indicating the successful preparation of CoMoO_4_ NSs on NiO flakes/NF. Moreover, no diffraction peaks of impurities are investigated for all samples, demonstrating the purity of the products.

XPS measurements were carried out to further determine the element component and chemical valence of the products. The survey spectrum displayed in Fig. 2b confirms the existence of Co, Mo, Ni, and O in the final products of NiO@CoMoO_4_/NF. As depicted in Fig. 2c, the high-resolution spectrum of Co 2p is divided into two major peaks at 781.3 eV and 797.4 eV, which can be fitted to Co 2p_3/2_ and Co 2p_1/2_, respectively [19]. Moreover, the two peaks located at the high binding energy side of major peaks are the corresponding satellite peaks. The Mo 3d spectrum in Fig. 2d is separated into two peaks of Mo 3d_5/2_ (232.2 eV) and Mo 3d_3/2_ (235.4 eV), indicating that Mo element exists in the form of Mo^6+^ oxidation state [20]. The high-resolution spectrum of Ni 2p (Fig. 2e) is clearly characterized by two peaks of Ni 2p_3/2_ and Ni 2p_1/2_ at the binding energies of 856.1 eV and 873.7 eV, respectively [21]. Similarly, the other two peaks located on high energy side are commonly considered as satellite peaks. As displayed in Fig. 2f, the high-resolution spectrum of O 1 s is divided into three oxidation states of O1, O2, and O3. O1 peak positioned at 530.7 eV can be attributed to lattice oxygen in the CoMoO_4_. O2 peak located at 531.5 eV is ascribed to metal-oxygen bond in NiO. O3 peak located at binding energy of 532.8 eV is associated with multiplicity molecular water adsorbed on the products [19]. Combined with XRD analysis, the results of XPS confirm the successful synthesis of NiO/CoMoO_4_ phase on NF.

**Fig. 3:**
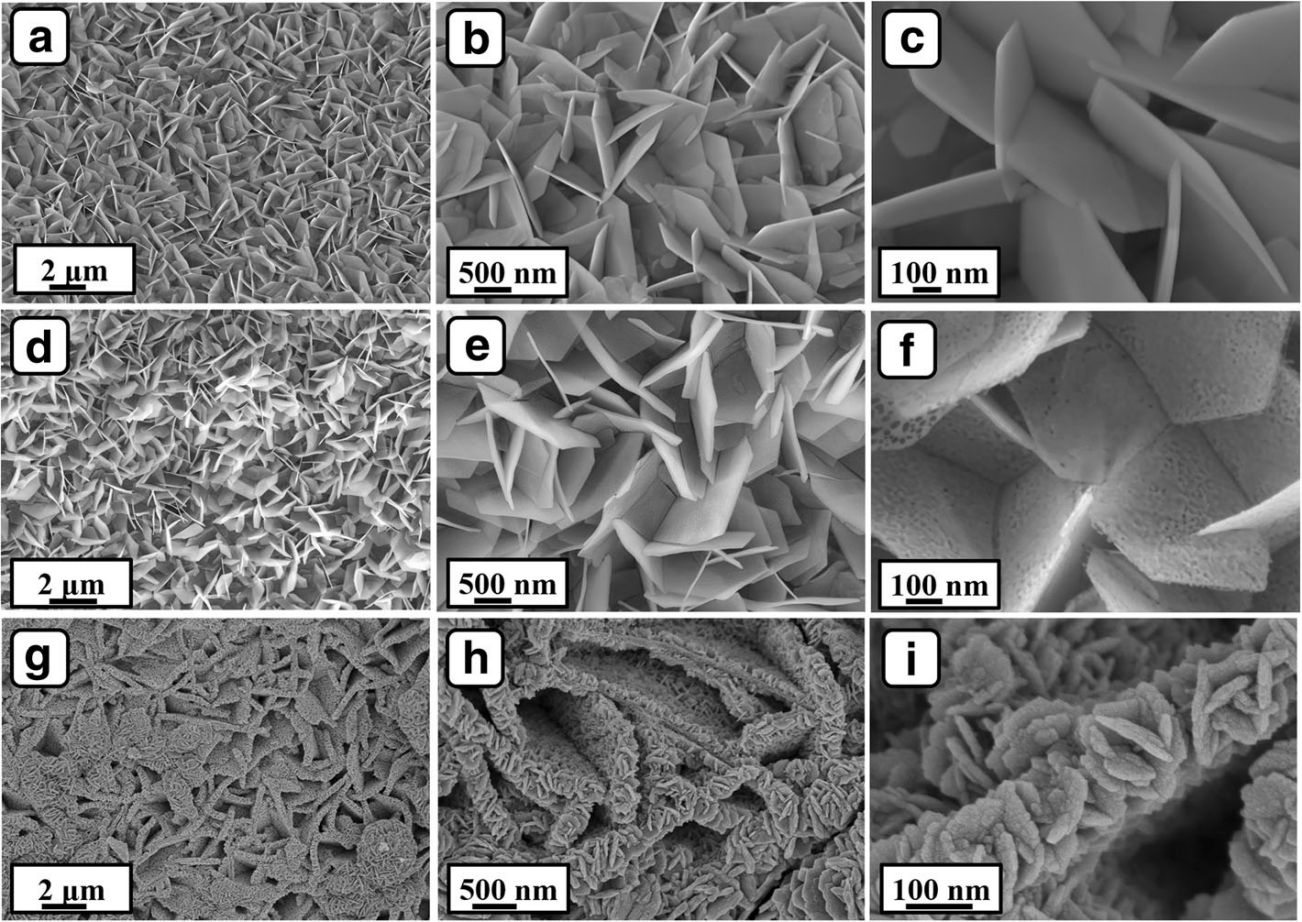
The SEM images of **a**–**c** Ni(OH)_2_ flakes/NF, **d**–**f** NiO flakes/NF, and **g**–**i** NiO flakes@CoMoO_4_ NSs/NF with different magnifications

As depicted in Fig. 3a, lots of Ni(OH)_2_ flakes were formed after the hydrothermal treatment of NF in distilled water. The flakes interact with each other and construct a 3D porous architecture. Hundreds of nanometers are clearly investigated between flakes, providing enough space for the further growth of CoMoO_4_ NSs (Fig. 3b). In Fig. 3c, the morphology of the flakes almost shows hexagonal feature with an edge length about 1–2 μm and a thickness of 30 nm. After heat treatment, the global morphology of the flakes has no significant change (Fig. 3d–f). However, the NiO flakes possess abundant pores on the surface (Fig. 3f), indicating mesoporous characteristic. The formed pores can be attributed to the loss of water in the heat treatment process. The porous structure possesses large specific surface area and accelerates the diffusion of electrolyte, benefiting the electrochemical kinetics [22]. After the second hydrothermal treatment, the thickness of the flakes apparently becomes thicker (Fig. 3g). Vast CoMoO_4_ NSs are deposited onto the both sides and top of the flakes (Fig. 3h), constructing a branched porous core-shell architecture. The 2D branched core-shell flakes have a width of 200–400 nm, which is much larger than that of NiO flakes. The CoMoO_4_ NSs have a width about 100 nm and thickness about 20–35 nm. The deposited CoMoO_4_ NSs afford more active sites for faraday reactions and promote the electronic collection and transfer rate, which may result in excellent capacitive performance. On the other hand, the size of CoMoO_4_ grown on NF (Additional file 1: Figure S1) is significantly larger than the size of CoMoO_4_ NSs on NiO flakes, proving that NiO flakes can coordinate the size of CoMoO_4_ flakes during the hydrothermal process.

**Fig. 4:**
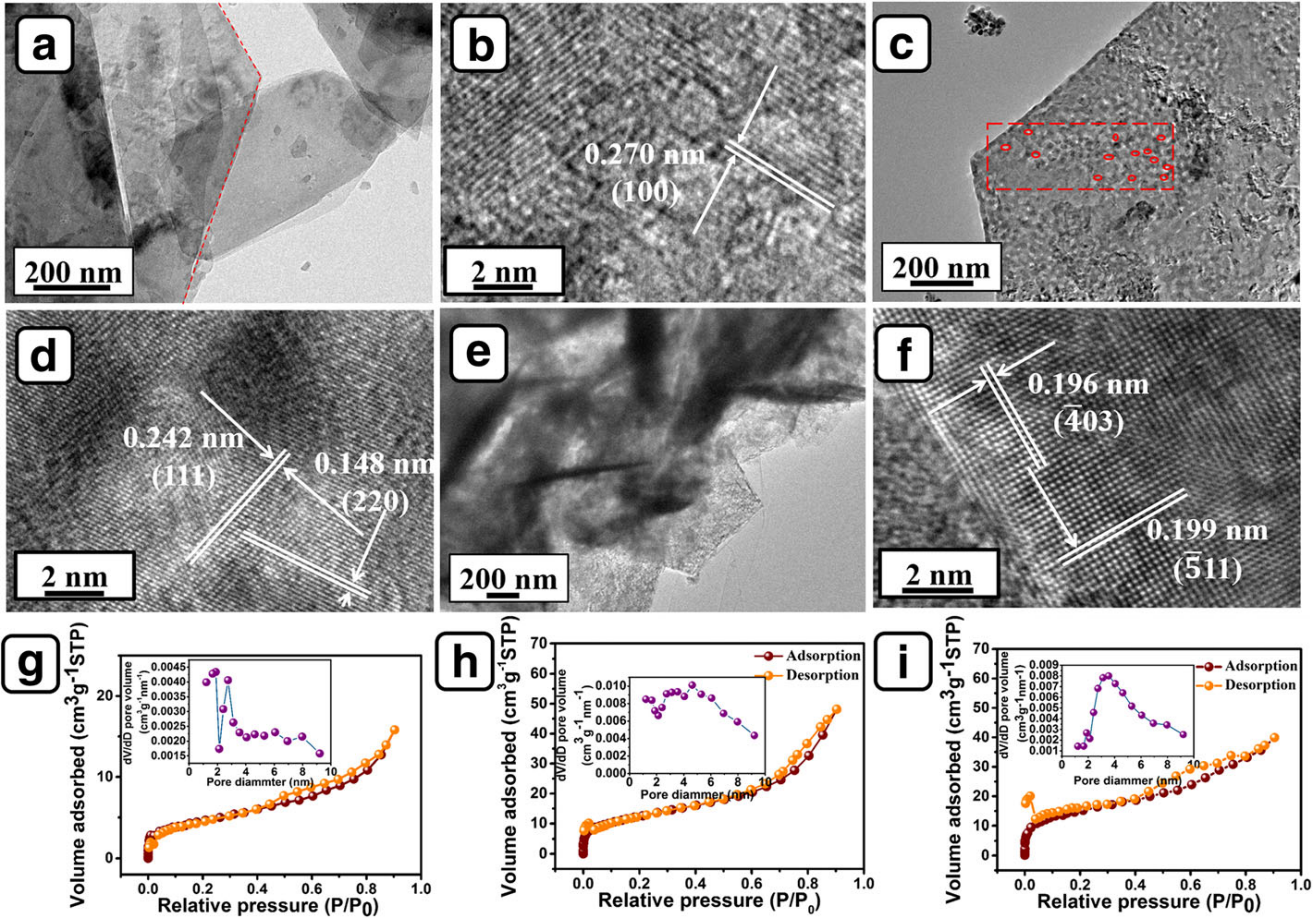
HRTEM images of **a**, **b** Ni(OH)_2_ flakes, **c**, **d** NiO flakes, **e**, **f** NiO flakes@CoMoO_4_ NSs; **g**–**i** are the nitrogen adsorption-desorption isotherms of Ni(OH)_2_ flakes/NF, NiO flakes/NF, and NiO flakes@CoMoO_4_ NSs/NF, respectively. Insets of (**g**–**i**) are the corresponding pore size distributions

In order to further research the morphology and structure of the products, different samples were stripped by ultrasound and investigated by HRTEM. As shown in Fig. 4a, Ni(OH)_2_ sample displays significant 2D feature. The lattice spacing observed in Fig. 4b (0.27 nm) corresponds to (100) plane of Ni(OH)_2_ (JCPDS No. 01-1047). After calcination, NiO sample still retains sheet-like morphology (Fig. 4c). Moreover, amounts of pores are clearly observed on the flakes. It is speculated that the formation of pores is caused by the loss of water. Figure 4d displays the lattice spacings of 0.242 nm and 0.148 nm, which can be attributed to the crystal plane of (111) and (220) of NiO (JCPDS No. 65-2901), respectively. The selected area electron diffraction (SAED) pattern demonstrates single-crystalline nature of the NiO flakes (Additional file 1: Figure S2a). From Fig. 4e, it is clear that CoMoO_4_ NSs are vertically grown on the surface of NiO flakes and the nanosheets show a thickness of 25–35 nm. The SAED pattern in Additional file 1: Figure S2b reveals polycrystalline feature of CoMoO_4_ flakes. The crystal lattice spacings measured in Fig. 4f (0.199 nm and 0.196 nm) are correlated to crystallographic plane ($\overline{4}$03) and ($\overline{5}$11) of CoMoO_4_, respectively (JCPDS No. 21-0868).

The N_2_ adsorption/desorption isothermal curves are commonly measured to judge the specific surface area and porosity of the products. As shown in Fig. 4g, the specific surface area of Ni(OH)_2_ flakes/NF is calculated as 28.2 m^2^/g, and the NiO flakes/NF obtained after calcination is 45.3 m^2^/g (Fig. 4h). The increase of the surface area is correlated to the formation of the pores on NiO flakes (Fig. 4c). Furthermore, the NiO flakes@CoMoO_4_ NSs/NF has a much larger value of 53.5 m^2^/g than NiO flakes/NF. The further increase of surface area can be attributed to the formation of diffusion channels constructed by CoMoO_4_ NSs. In addition, all the N_2_ adsorption/desorption isothermal curves belong to type of IV hysteresis, demonstrating mesoporous feature of the products [23–25]. The mean pore diameters of Ni(OH)_2_ flakes/NF, NiO flakes/NF, and NiO flakes@CoMoO_4_ NSs/NF are 6.13 nm, 6.57 nm, and 4.16 nm, respectively. The larger specific surface area and smaller pores distribution are beneficial for the increase of active sites and the promotion of electrolyte diffusion, leading to enhanced electrochemical performance [22].

### The Electrochemical Performance of NiO@CoMoO_4_/NF

The electrochemical performance of NiO flakes@CoMoO_4_ NSs/NF is evaluated as a positive electrode for supercapacitor. The calculation formula of specific capacitance (C_s_) is displayed in Eq. (3) [26]:

3 $$ {C}_s=\frac{i\varDelta t}{mV} $$

Where *m* is the mass of active materials, *V* is the potential window, *i* is the current, and *∆t* is the discharge time.

**Fig. 5:**
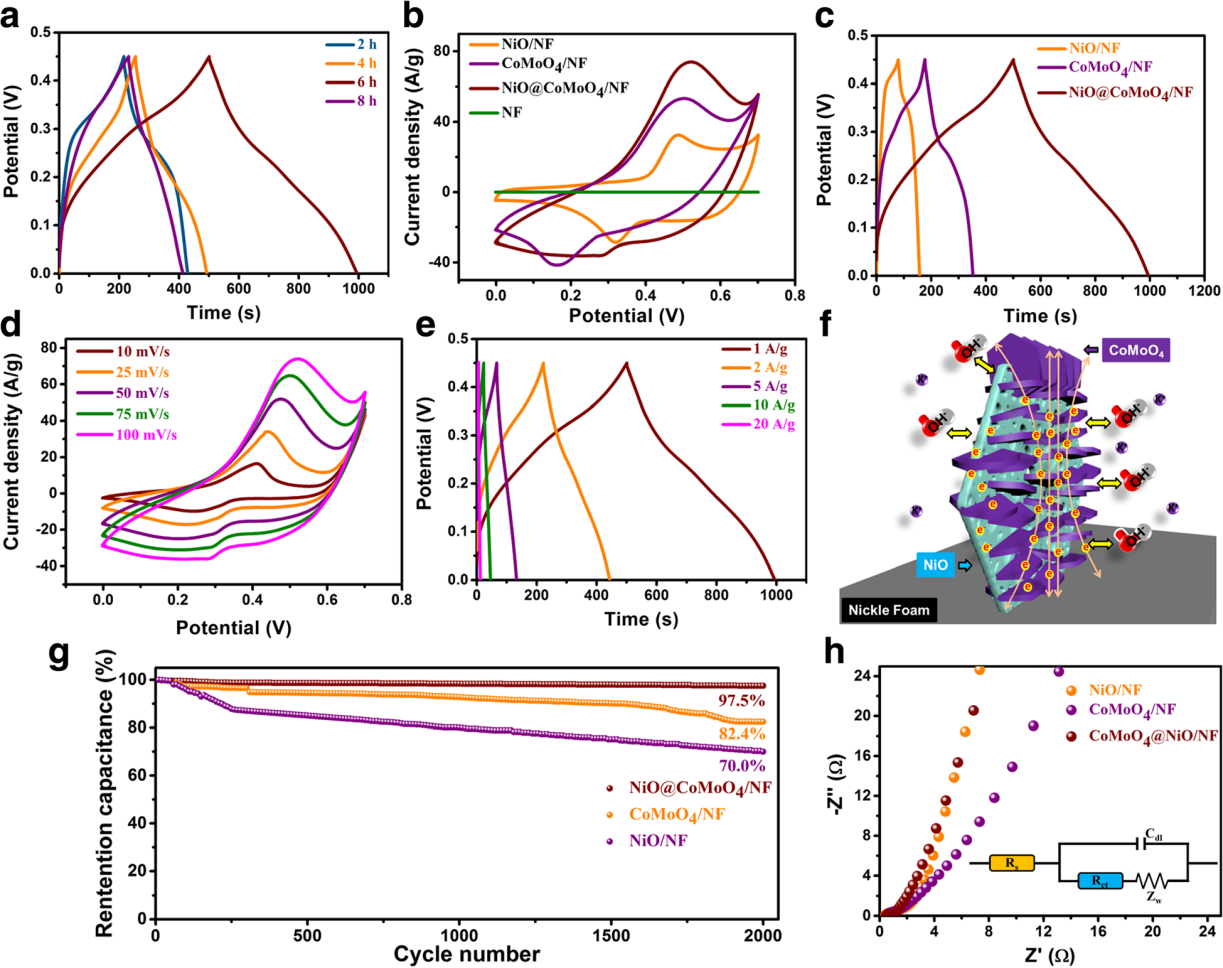
**a** GCD curves of NiO flakes@CoMoO_4_ NSs/NF electrodes obtained at different reaction time (2 h, 4 h, 6 h, and 8 h) at current density of 1 A/g. **b** CV curves of NiO flakes/NF, CoMoO_4_ flakes/NF, and NiO flakes@CoMoO_4_ NSs/NF electrodes at scan rate of 100 mV/s. **c** GCD curves of NiO flakes/NF, CoMoO_4_ flakes/NF, and NiO flakes@CoMoO_4_ NSs/NF electrodes at current density of 1 A/g. **d** CV curves of NiO flakes@CoMoO_4_ NSs/NF electrode at different scan rates. **e** GCD curves of NiO flakes@CoMoO_4_ NSs/NF electrode at different current densities. **f** The schematic diagram of structural advantages for NiO flakes@CoMoO_4_ NSs/NF. **g** Cycle stability of different electrodes up to 2000 cycles. **h** EIS spectra of NiO flakes/NF, CoMoO_4_ flakes/NF, and NiO flakes@CoMoO_4_ NSs/NF electrodes

In order to achieve better capacitive performance, NiO flakes@CoMoO_4_ NSs/NF obtained at different reaction time (2 h, 4 h, 6 h, 8 h) were measured by GCD at 1 A/g. As can be seen from Fig. 5a, the specific capacitance increases with the increasing of reaction time until 6 h. However, the specific capacitance sharply decreases when the reaction time reaches 8 h. Moreover, the GCD curves of the different samples (2 h, 4 h, and 8 h) are shown in Additional file 1: Figure S3. Combined with the morphology observations shown in Additional file 1: Figure S4, the initial increase of specific capacitance can be attributed to the mass increase of CoMoO_4_ NSs and the construction of 2D branched core-shell architecture on the surface of NiO flakes. When the reaction time reaches 8 h, the branched core-shell architecture is almost covered by the tiny CoMoO_4_ NSs, leading to difficulties in electrochemical kinetics. Thus, the product obtained at 6 h presents the best capacitive performance. Additionally, the selectivity of 160 °C was also discussed in Additional file 1: Figure S5.The electrochemical performance of NiO flakes@CoMoO_4_ NSs/NF (6 h) was further researched compared to NiO flakes/NF and CoMoO_4_ flakes/NF. The CV curves of NiO flakes@CoMoO_4_ NSs/NF (6 h), NiO flakes/NF, and CoMoO_4_ flakes/NF were displayed in Fig. 5b. It is well known that the encapsulated area of CV curves is proportional to the specific capacitance of electrode materials. As shown in Fig. 5b, the CV encapsulated area of NF can be ignored compared to other three electrodes, indicating little contribution of NF. The CV curve of NiO flakes@CoMoO_4_ NSs/NF (6 h) shows largest encapsulated area compared to NiO flakes/NF and CoMoO_4_ flakes/NF, demonstrating highest specific capacitance. Similarly, the CoMoO_4_ flakes/NF electrode presents higher specific capacitance than NiO flakes/NF. As depicted in Fig. 5c, the symmetric GCD curve and longer discharge time of NiO flakes@CoMoO_4_ NSs/NF electrode testify outstanding coulomb efficiency and higher specific capacitance compared to other two electrodes under 1 A/g. In addition, CoMoO_4_ flakes/NF electrode presents longer discharge time than NiO flakes/NF electrode, demonstrating higher specific capacitance. The results of Fig. 5c is consistent with the analysis of CV curves. Figure 5d displays the CV curves of NiO flakes@CoMoO_4_ NSs/NF (6 h) at different scan rates. Apparently, redox peaks are observed in the series of CVs, revealing pseudocapacitive characteristic of NiO flakes@CoMoO_4_ NSs/NF (6 h). The CV curve still retains well-defined outline under high scan rate, demonstrating high-efficiency ionic and electron transfer rate. Accordingly, the CV curves of NiO flakes/NF and CoMoO_4_ flakes/NF also display typical pseudocapacitive feature (Additional file 1: Figure S6a, b). The charge storage mechanism can be associated with the redox of metal composition in alkaline solution [27, 28]:

$$ {\displaystyle \begin{array}{l}3{\left[\mathrm{Co}{\left(\mathrm{OH}\right)}_3\right]}^{-}\leftrightarrow {\mathrm{Co}}_3{\mathrm{O}}_4+4{\mathrm{H}}_2{\mathrm{O}\mathrm{H}}^{\hbox{-} }+2{\mathrm{e}}^{\hbox{-}}\\ {}{\mathrm{Co}}_3{\mathrm{O}}_4\kern0.5em +\kern0.5em {\mathrm{H}}_2\mathrm{O}\kern0.5em +\kern0.5em {\mathrm{O}\mathrm{H}}^{\hbox{-}}\leftrightarrow 3\mathrm{CoOOH}\kern0.5em +\kern0.5em {e}^{-}\\ {}\mathrm{CoOOH}\kern0.5em +\kern0.5em {\mathrm{O}\mathrm{H}}^{-}\leftrightarrow {\mathrm{Co}\mathrm{O}}_2+{\mathrm{H}}_2\mathrm{O}+{e}^{-}\\ {}\mathrm{NiO}\kern0.5em +\kern0.5em {\mathrm{O}\mathrm{H}}^{-}\leftrightarrow \mathrm{NiOOH}\kern0.5em +\kern0.5em {\mathrm{e}}^{-}\end{array}} $$

The specific capacitance of NiO flakes@CoMoO_4_ NSs/NF is mainly derived from the quasi-reversible redox of Co^2+^/Co^3+^ and Ni^2+^/Ni^3+^, and Mo is not involved into redox reaction. Furthermore, the peak current of CV is linearly increased with the sweep rate, illustrating that the typical polarization of the electrode controls the electrochemical kinetic process [29]. The GCD curves of NiO flakes@CoMoO_4_ NSs/NF electrode at different charge-discharge currents are shown in Fig. 5e. The NiO flakes@CoMoO_4_ NSs/NF electrode presents specific capacitances of 1097 F/g, 981 F/g, 734 F/g, 504 F/g, and 262 F/g at current densities of 1 A/g, 2 A/g, 5 A/g, 10 A/g, and 20 A/g, respectively. Accordingly, the corresponding specific capacitances of CoMoO_4_ flakes/NF (Additional file 1: Figure S6c) and NiO flakes/NF (Additional file 1: Figure S6d) are 349 F/g, 316 F/g, 248 F/g, 182 F/g, 116 F/g, and 173 F/g, 160 F/g, 139 F/g, 116 F/g, 80 F/g, respectively. Apparently, NiO flakes@CoMoO_4_ NSs/NF electrode presents higher specific capacitance than the individual NiO flakes/NF and CoMoO_4_ flakes/NF electrodes, demonstrating synergistic effect between NiO flakes and CoMoO_4_ NSs. As illustrated in Fig. 5f, the synthesized 2D branched of NiO flakes@CoMoO_4_ NSs/NF composite provides beneficial kinetics conditions in terms of effective ions transport channels, short ion diffusion distance, fast charge transfer rate, and plentiful redox active sites, resulting in excellent capacitive performance [30].

The cycle life as one of the key factors for supercapacitors was measured by performing 2000 cycles GCD at current density of 2 A/g. Figure 5g demonstrates that the NiO flakes@CoMoO_4_ NSs/NF electrode still retains 97.5% of original specific capacitance. However, the specific capacitance of NiO flakes/NF and CoMoO_4_ flakes/NF electrodes decrease to 82.4% and 70% of their original capacitance, respectively. The 2D branched porous structure provides enough spaces, which are beneficial for the effective stress relaxation of volume change during the cycle process, resulting in excellent cycling stability.

Finally, the EIS spectra of the researched electrodes were measured and the equivalent circuit diagram was illustrated as an inset. As shown in Fig. 5h, all the spectra display an obvious semicircle at high frequency and a linear region in low-frequency range. The intersection with the *x*-axis and semicircle radius represent the equivalent series resistance (*R*_*s*_) and charge transfer resistance (*R*_*ct*_) on electrode interface, respectively. The slope of linear region corresponds the mass diffusion resistance (*Z*_*w*_). As shown in Additional file 1: Table S1, the NiO flakes@CoMoO_4_ NSs/NF electrode has lower *R*_*s*_ and *R*_*ct*_ (0.4 Ω, 0.21 Ω) than those of CoMoO_4_ flakes/NF (0.58 Ω, 0.93 Ω) and NiO flakes/NF (0.48 Ω, 0.72 Ω). Obviously, the NiO@CoMoO_4_/NF electrode presents significant advantages in electron transfer kinetics, demonstrating potential applications as an ideal electrode material for supercapacitors.

### The Performance of NiO Flakes@CoMoO_4_ NSs/NF//AC/NF

**Fig. 6:**
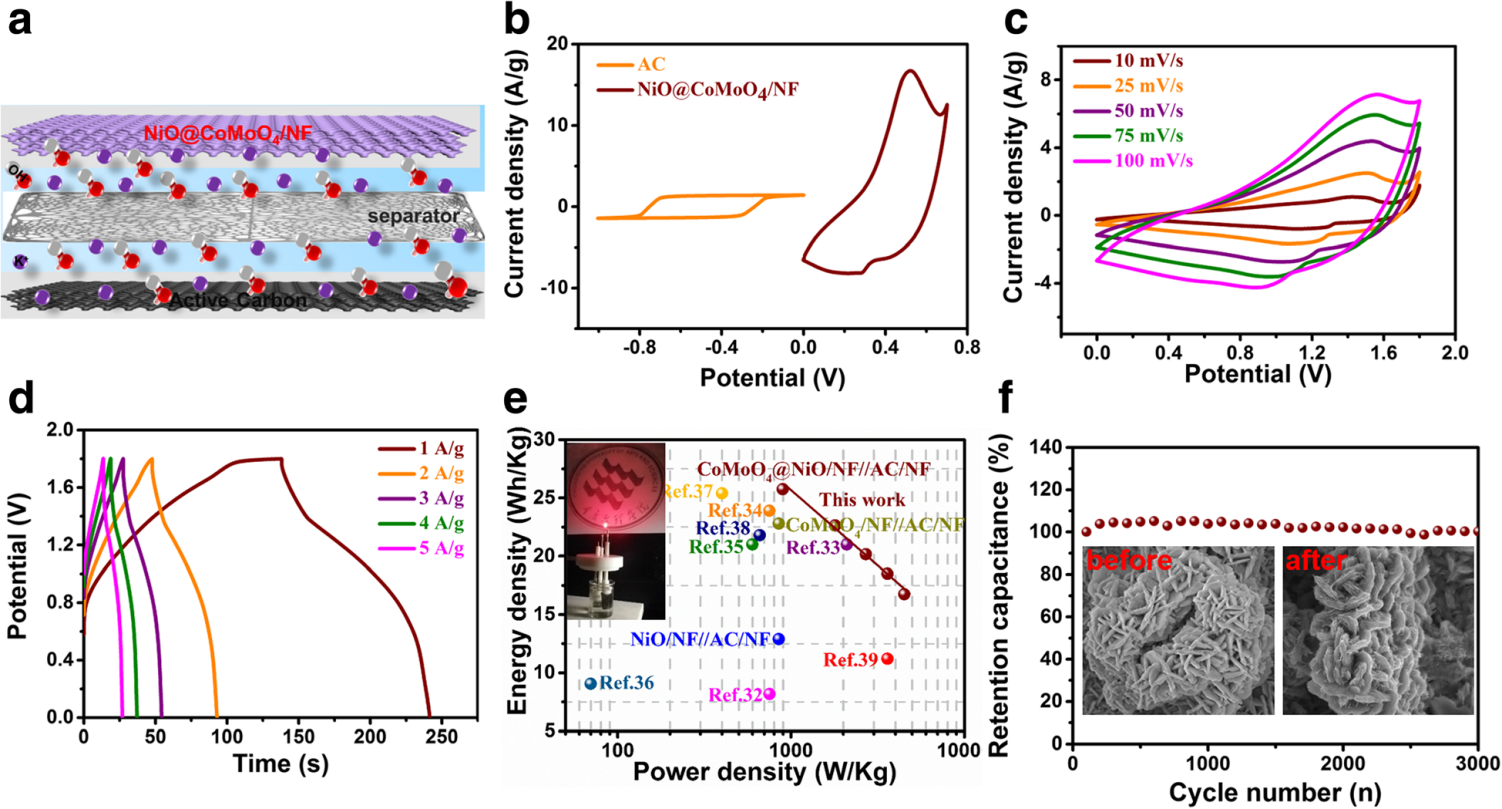
**a** The structure illustration of the ASC device. **b** CV curves of NiO flakes@CoMoO_4_ NSs/NF and AC in three-electrode system. **c** CV curves of the ASC device at different scan rates. **d** GCD curves of the ASC device at different current densities. **e** Ragone plots of the ASC and the comparation with other reported NiO or CoMoO_4_ electrodes. **f** Cycling stability of the ASC device over 3000 cycles at a current density of 5 A/g. Inset is the SEM images before and after cycling

To demonstrate the practical applications of NiO flakes@CoMoO_4_ NSs/NF composite electrode, an ASC was assembled in 6 M KOH according to the illustration in Fig. 6a. In the ASC, NiO flakes@CoMoO_4_ NSs/NF was used as the positive electrode paired with commercial AC as the negative electrode. As can be seen from the CV measurements in Fig. 6b, the AC electrode presents rectangular feature and the NiO flakes@CoMoO_4_ NSs/NF electrode displays significant redox peaks, revealing typical electrochemical storage mechanisms of EDLC and pseudocapacitive, respectively. Furthermore, a potential window as high as 1.8 V can be achieved through the combination of positive and negative electrodes. The CV curves of NiO flakes@CoMoO_4_ NSs/NF//AC/NF ASC at different scan rates were plotted in Fig. 6c. The ASC still can be cycled with well-defined shape even at high scan rate, indicating beneficial kinetics in electron transfer and ionic transport. GCD curves of the ASC at different current densities from 1 to 5 A/g were recorded in Fig. 6d. The energy density and power density of the ASC were calculated by following Eqs. (4) and (5), respectively [31]:

4 $$ E=\frac{1}{2\times 3.6}{C}_s\varDelta {V}^2 $$

5 $$ P=\frac{E\times 3600}{\varDelta t} $$

Where *E* is the energy density, *P* is the power density, *C*_*s*_ is the specific capacitance, *ΔV* is the potential window, and *Δt* is the discharge time. As shown in the Ragone plot (Fig. 6e), the NiO flakes@CoMoO_4_ NSs/NF//AC/NF ASC presents a maximum energy density of 25.8 Wh/kg at power density of 894.7 W/kg and a high energy density of 16.8 Wh/kg is still retained even at high power density of 4500 W/kg. As displayed in the inset of Fig. 6e, a single red LED was lighted and lasted 10 min by 1.93 mg (1 cm × 1 cm) electrode materials. The maximum energy density is higher than the individual NiO/NF//AC/NF (12.9 Wh/kg, Additional file 1: Figure S7a) and CoMoO_4_ flakes/NF//AC/NF (22.8 Wh/kg, Additional file 1: Figure S7b), further confirming the synergistic effect between NiO flakes and CoMoO_4_ NSs. Compared with other NiO or CoMoO_4_-based electrodes, the NiO@CoMoO_4_/NF//AC/NF ASC exhibits higher energy density [32–39]. The cycle life of the ASC was evaluated by repeating GCD measurement at 5 A/g for 3000 cycles. As shown in Fig. 6f, the capacitance retains 100% compared with its original value after 3000 cycles. As shown in the inset of Fig. 6f, the morphology structure presents little difference before and after the cycling, demonstrating excellent cycle stability of the electrode materials.

## Conclusion

In summary, NiO flakes@CoMoO_4_ NSs core-shell architecture was successfully fabricated by a two-step hydrothermal method. As a positive electrode for supercapacitors, NiO flakes@CoMoO_4_ NSs/NF electrode exhibits remarkable electrochemical properties, including high specific capacitance of 1097 F/g, low charge transfer resistance of 0.21 Ω, and excellent long-term cycling stability (retains 97.5% of its original value after 2000 cycles). The high specific surface area, effective ions transport channels, and accelerated electron collect/transfer rate are responsible for the prominent electrochemical performance. The assembled ASC device exhibits a distinguished energy density of 25.8 Wh/kg at power density of 894.7 W/kg. Simultaneously, the ASC device retains 100% of its original specific capacitance after 3000 cycles, demonstrating excellent cycling stability. The NiO flakes@CoMoO_4_ NSs/NF electrode has promising prospects in supercapacitors and the design of 2D branched core-shell architecture paves an effective way to achieve high-performance electrode materials for energy storage.

## Additional File

Additional file 1: **Figure S1.** SEM and TEM images of CoMoO_4_ flakes/NF **Figure S2.** SAED patterns of (a) NiO flakes/NF and (b) CoMoO_4_ flakes/NF **Figure S3.** GCD curves of the samples obtained at different reaction time. (a) 2 h; (b) 4 h; (c) 8 h.** Figure S4.** SEM images of the NiO flakes@CoMoO_4_ NSs/NF obtained at different reaction time** Figure S5.** SEM images of NiO flakes@CoMoO_4_ nanosheets obtained at (a) 120 °C, (b) 140 °C, (c) 160 °C and (d) 180 °C; (e) GCD curves at 1 A/g of NiO flakes@CoMoO_4_ nanosheets obtained at different temperatures **Figure S6.** CV curves and GCD curves of (a, c) NiO flakes/NF and (b, d) CoMoO_4_ flakes/NF **Figure S7.** GCD curves of (a) NiO flakes/NF//AC/NF and (b) CoMoO_4_ flakes/NF//AC/NF. **Table S1.** Fitting of Nyquist plots for the researched three electrodes (DOCX 6402 kb)

## Data Availability

All data are fully available without restriction.

## Abbreviations

2D: Two-dimensional
AC: Active carbon
ASC: Asymmetric supercapacitor
CV: Cyclic voltammetry
EDLCs: Electric double layer capacitors
EIS: Electrochemical impedance spectroscopy
EQ: Equation
FESEM: Field emission scanning electron microscopy
GCD: Galvanostatic charge-discharge
HRTEM: High-resolution transmission electron microscope
NF: Ni foam
NSs: Nanosheets
PTFE: Polytetrafluoroethylene
TMOs: Transition metal oxides
XPS: X-ray photoelectron spectroscopy
XRD: X-ray diffractometer
