# Supplementary material for: Design of NiO Flakes@CoMoO4 Nanosheets Core-Shell Architecture on Ni Foam for High-Performance Supercapacitors
Source: Nanoscale Res Lett. 2019 Jul 2;14:221. doi: 10.1186/s11671-019-3054-3 (PMC6606686; doi:10.1186/s11671-019-3054-3)
Supplement: Supplementary file 1 — Figure S1. SEM and TEM images of CoMoO4 flakes/NF Figure S2. SAED patterns of (a) NiO flakes/NF and (b) CoMoO4 flakes/NF Figure S3. GCD curves of the samples obtained at different reaction time. (a) 2 h; (b) 4 h; (c) 8 h. Figure S4. SEM images of the NiO flakes@CoMoO4 NSs/NF obtained at different reaction time Figure S5. SEM images of NiO flakes@CoMoO4 nanosheets obtained at (a) 120 °C, (b) 140 °C, (c) 160 °C and (d) 180 °C; (e) GCD curves at 1 A/g of NiO flakes@CoMoO4 nanosheets obtained at different temperatures Figure S6. CV curves and GCD curves of (a, c) NiO flakes/NF and (b, d) CoMoO4 flakes/NF Figure S7. GCD curves of (a) NiO flakes/NF//AC/NF and (b) CoMoO4 flakes/NF//AC/NF. Table S1. Fitting of Nyquist plots for the researched three electrodes (DOCX 6402 kb) [file 11671_2019_3054_MOESM1_ESM.docx]

Design of NiO flakes@CoMoO_4_ nanosheets core-shell architecture on Ni foam for high-performance supercapacitors

Supporting information

Enmin Zhou^1, 2^, Liangliang Tian^1, *^, Zhengfu Cheng^1, *^, Chunping Fu^1^

^1^Research Institute for New Materials Technology, Chongqing University of Arts and Sciences, Chongqing, PR China

^2^School of Science, Chongqing University of Posts and Telecommunications, Chongqing, PR China

Email addresses: [tian](mailto:chzhfu8@163.com)ll07@163.com

**This document file includes:**

Supplementary Figure S1 to S6

Table S1


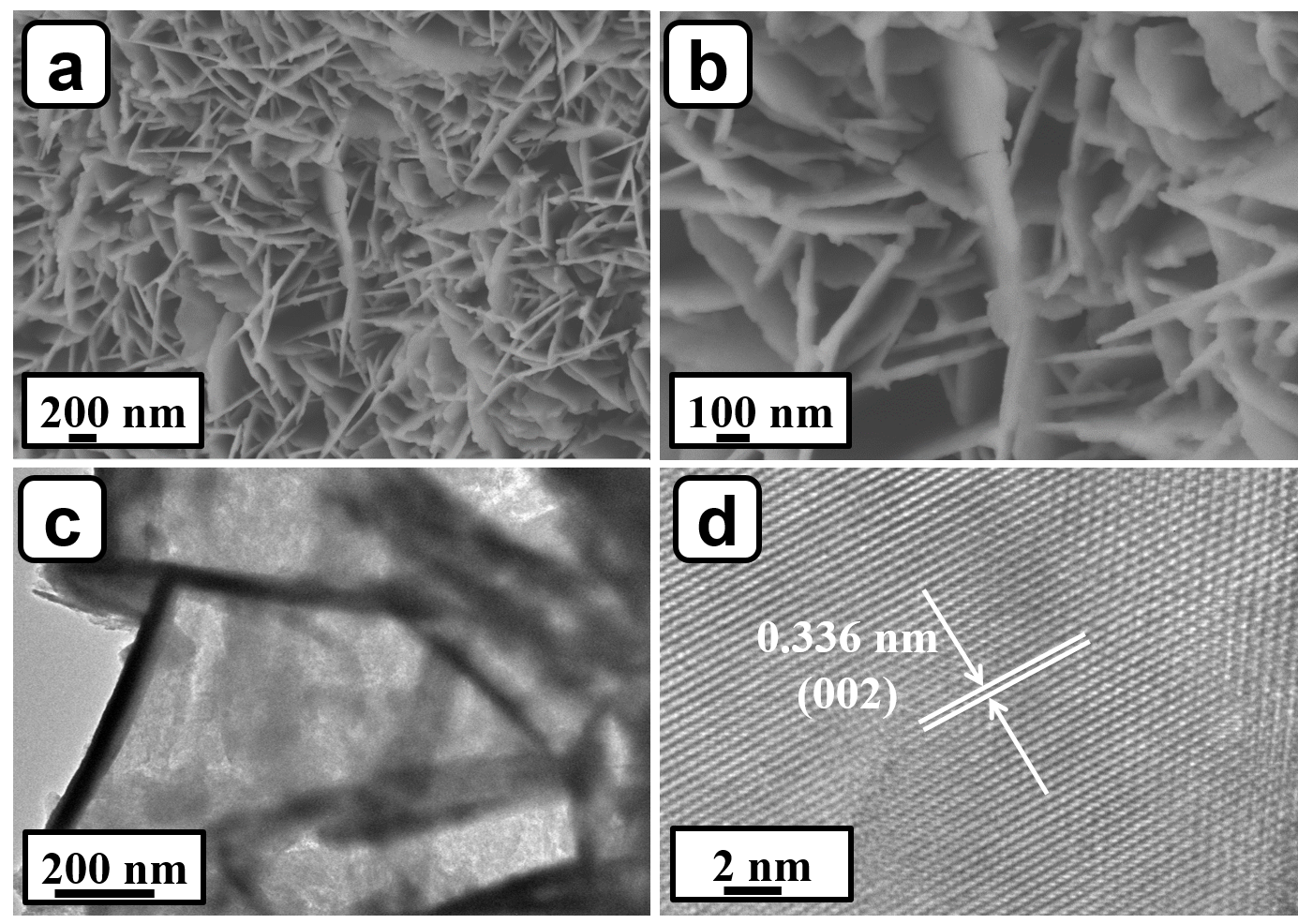


Figure S1. SEM and TEM images of CoMoO_4_ flakes/NF


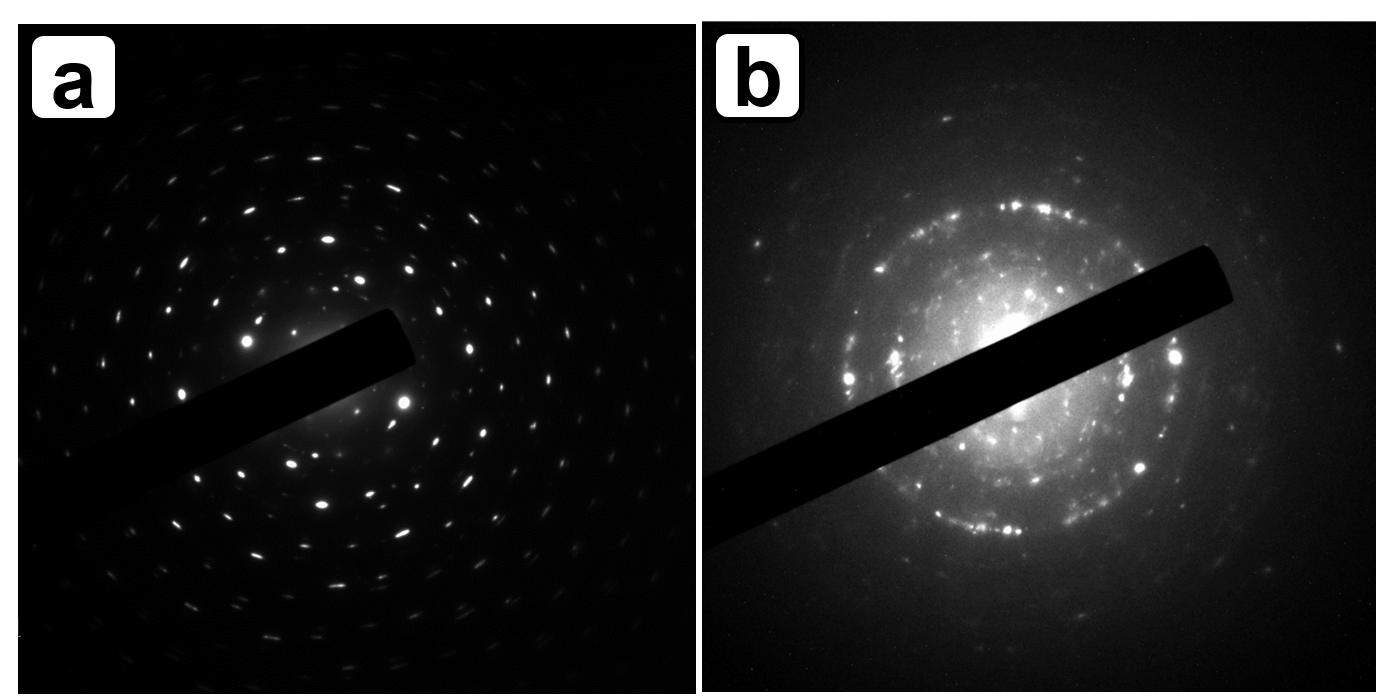


Figure S2. SAED patterns of (a) NiO flakes/NF and (b) CoMoO_4_ flakes/NF.


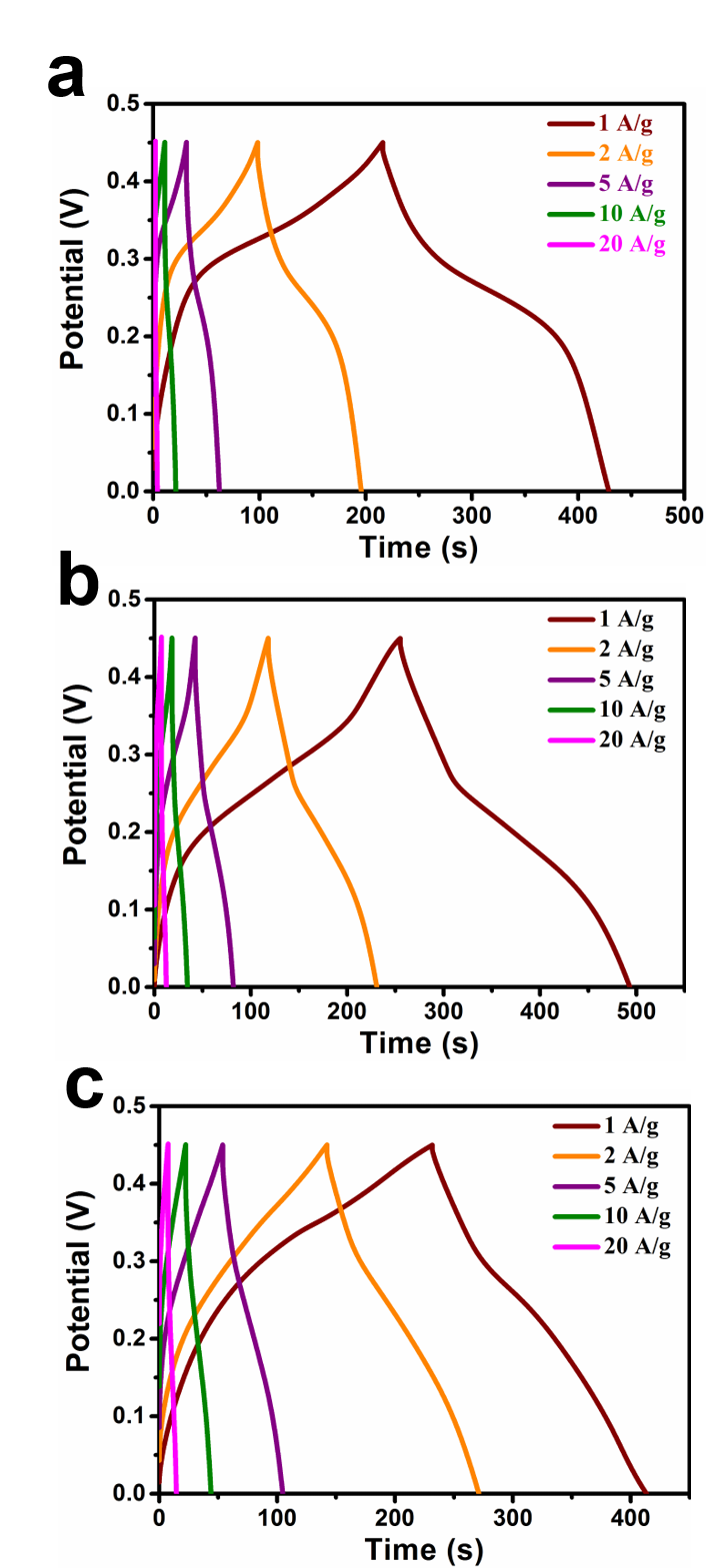


Figure S3. GCD curves of the samples obtained at different reaction time. (a) 2 h; (b) 4 h; (c) 8 h.


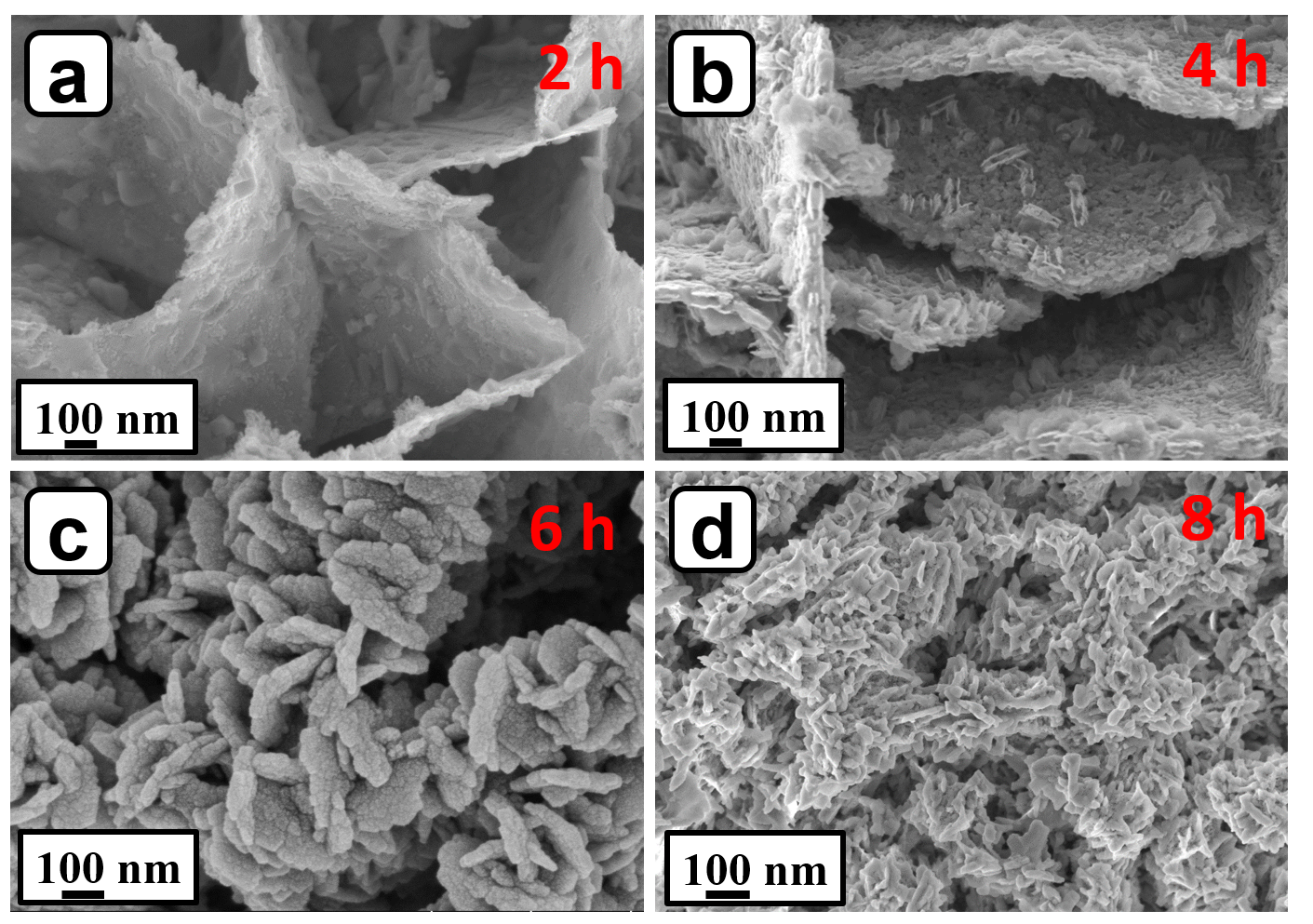


Figure S4. SEM images of the NiO flakes@CoMoO_4_ NSs/NF obtained at different reaction time


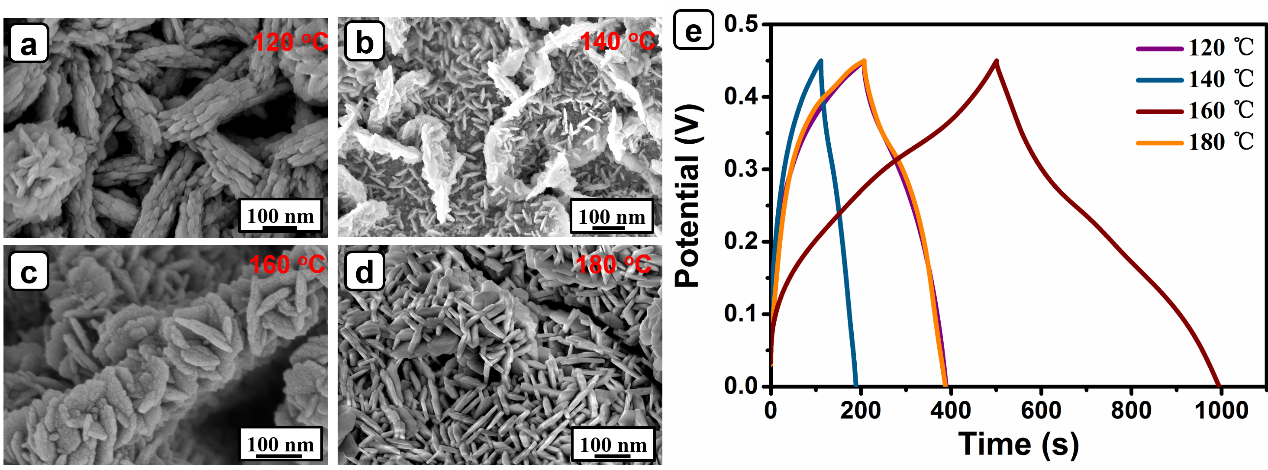


Figure S5. SEM images of NiO flakes@CoMoO_4_ nanosheets obtained at (a) 120 ^o^C, (b) 140 ^o^C, (c) 160 ^o^C and (d) 180 ^o^C; (e) GCD curves at 1 A/g of NiO flakes@CoMoO_4_ nanosheets obtained at different temperatures.


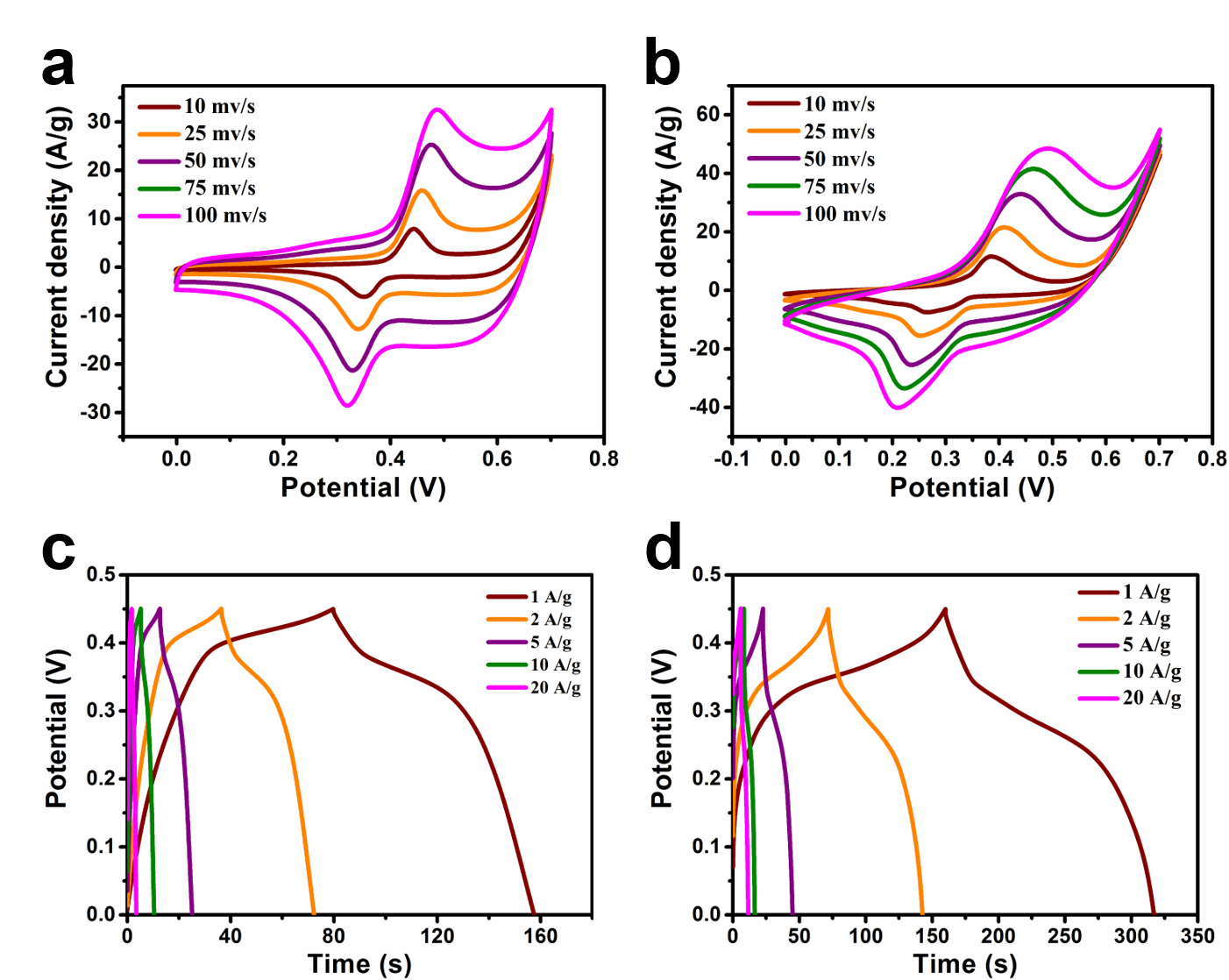


Figure S6. CV curves and GCD curves of (a, c) NiO flakes/NF and (b, d) CoMoO_4_ flakes/NF.


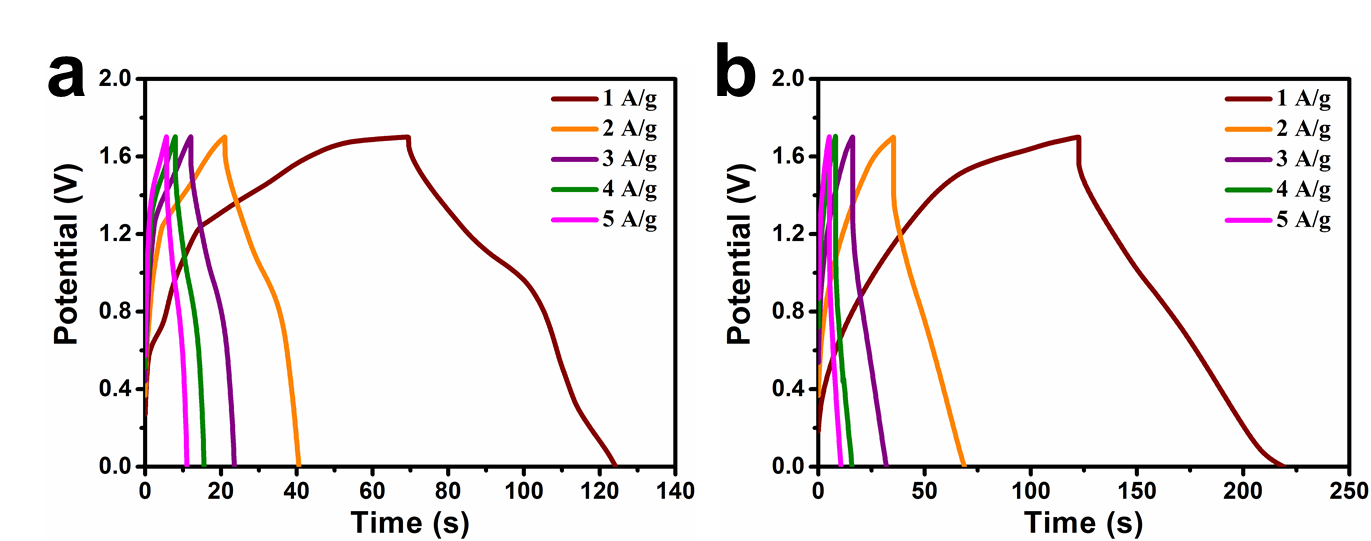


Figure S7. GCD curves of (a) NiO flakes/NF//AC/NF and (b) CoMoO_4_ flakes/NF//AC/NF.

Table S1 Fitting of Nyquist plots for the researched three electrodes

| Electrode materials | *R_s_* | *R_ct_* |
| --- | --- | --- |
| NiO/NF | 0.48 Ω | 1.24 Ω |
| CoMoO_4_/NF | 0.58 Ω | 0.93 Ω |
| NiO@CoMoO_4_/NF | 0.40 Ω | 0.21 Ω |
